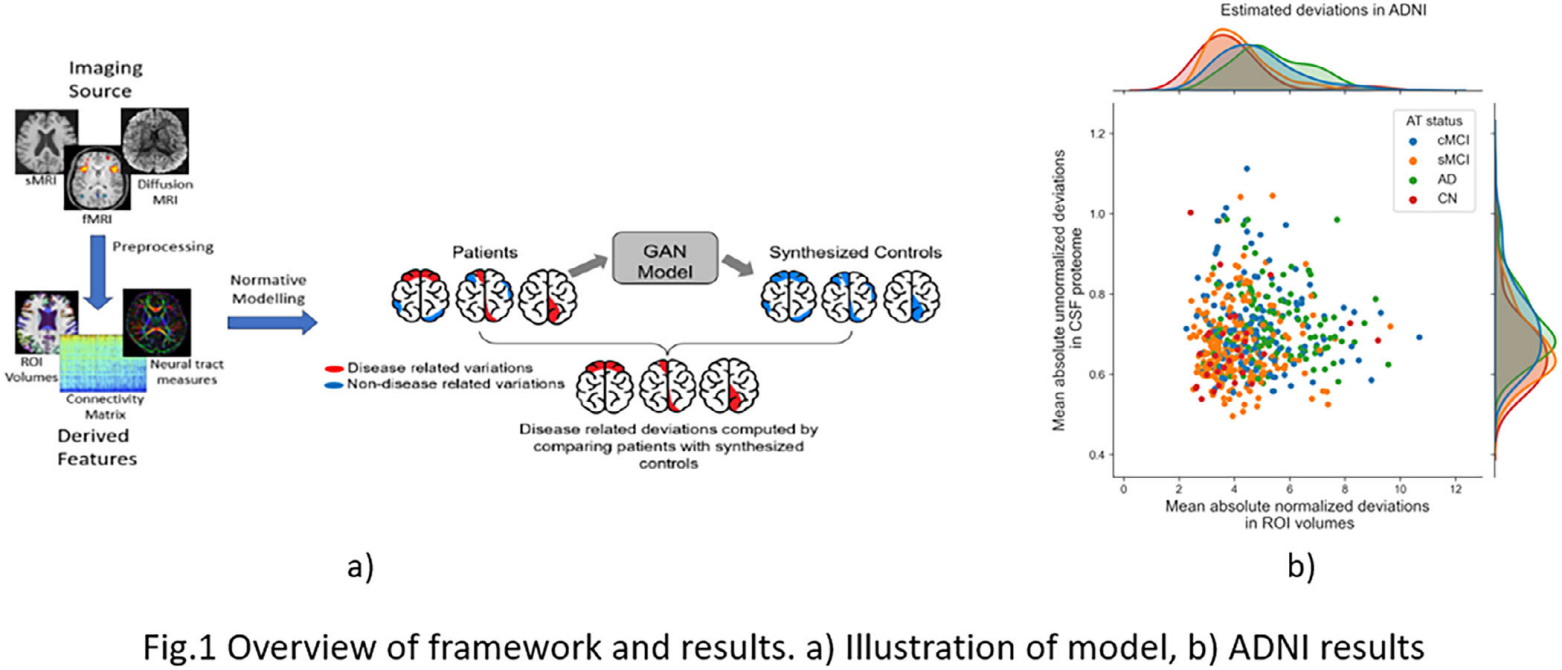# Multivariate normative Modeling of structural brain regional volumes and CSF‐based proteomics to investigate personalized effects related to Alzheimer's Disease

**DOI:** 10.1002/alz70856_102126

**Published:** 2025-12-25

**Authors:** Sai Spandana Chintapalli

**Affiliations:** ^1^ University of Pennsylvania, Philadelphia, PA, USA

## Abstract

**Background:**

Pathological heterogeneity in patients with Alzheimer's disease (AD) leads to diagnostic and prognostic uncertainty and confounds clinical treatment planning. Normative modeling, where individual‐level deviations in biological features from a reference sample are computed to infer personalized effects of disease, allows parsing of disease heterogeneity. In this study, GAN‐based normative modeling technique quantifies individual‐level neuroanatomical and proteomic abnormality thereby facilitating measurement of personalized disease‐related effects in AD patients.

**Method:**

We adapt the pix2pix GAN to translate a subject with disease to a corresponding subject without disease. To identify disease effects, we train this model using healthy controls (CN) and synthetically simulated patients. Model detecting neuroanatomical heterogeneity is trained using MRI‐based region of interest (ROI) volume data from CN (*n* = 6000) selected from the ISTAGING consortium. Model detecting proteomic heterogeneity is trained using CSF‐based protein expression data from CN (*n* = 96) selected from the ADNI study. The two models select 139 ROI volumes (gray and white matter structures) computed using a multi‐atlas segmentation technique and 279 brain‐enriched proteins as biological measures, respectively. Patients are simulated by perturbing the reference sample (Figure 1a). Deviation of the patient from the synthesized disease‐free control acts as a personalized biomarker that is sensitive to disease effects and severity. For performance assessment, we select 517 held‐out participants (31 CN, 220 MCI that remain stable (sMCI), 139 MCI that convert to AD (cMCI), 128 AD) from the ANDI dataset and compute their structural and proteomic deviations using the respective models. Mean absolute deviation (aggregate deviation across selected features) is used to infer pathology‐related effects.

**Result:**

Larger deviations in cMCI and AD compared to CN and sMCI suggest disease related abnormality in both CSF proteomic and structural ROI measures (Figure 1b).

**Conclusion:**

GAN‐based normative modeling technique introduced here is a useful tool to parse heterogeneity at an individual level. The model detects pathology effects in both structural and molecular data, highlighting its potential at personalized deviation detection.